# Internet-based group compassion-focused therapy for Swedish young people with stress, anxiety and depression: a pilot waitlist randomized controlled trial

**DOI:** 10.3389/fpsyg.2025.1547046

**Published:** 2025-04-01

**Authors:** Magnus Vestin, Linda Wallin, Matilda Naesström, Ida Blomqvist, Carl Göran Svedin, Elaine Beaumont, Jussi Jokinen, Inga Dennhag

**Affiliations:** ^1^Department of Clinical Science, Child and Adolescent Psychiatry, Umeå University, Umeå, Sweden; ^2^Department of Clinical Sciences/Psychiatry, Umeå University, Umeå, Sweden; ^3^Department of Social Sciences, Marie Cederschiöld University, Stockholm, Sweden; ^4^School of Health and Society, University of Salford, Salford, United Kingdom; ^5^Department of Clinical Neuroscience, Karolinska Institutet, Stockholm, Sweden

**Keywords:** compassion, young people, stress, randomized controlled trial (RCT), group psychotherapy, internet-based psychotherapy

## Abstract

**Introduction:**

Compassion-focused therapy (CFT) has shown promising outcomes for young people, but research on CFT for this population remains limited. This study aims to evaluate the feasibility and acceptability of a seven-session, therapist-led, internet-based group CFT for young people, and to investigate its preliminary effects.

**Methods:**

A two-arm pilot randomized controlled trial (RCT) was conducted. The study included 42 participants (aged 15–20), experiencing mild to moderate stress, anxiety, or depression, most of whom (90%) were female. In the intervention group, 22 participants were included in the intention-to-treat (ITT) analysis. The trial was registered at ClinicalTrials.gov (NCT05448014).

**Results:**

The intervention group had low attrition and moderate attendance, with 77% completing four or more modules. No adverse events were reported, and participants generally expressed satisfaction with the intervention. Linear regression models showed preliminary between-group differences in two variables. Depressive symptoms increased post-intervention for individuals in the intervention group compared to the waitlist (WL) group (*p* = 0.002). Self-compassion improved in the intervention group (*p* = 0.023). These patterns were consistent among participants who completed more than two sessions. Within-group analyses indicated moderate, significant improvements in stress, self-compassion and compassion from others.

**Discussion:**

These preliminary results suggest that CFT is feasible and acceptable and may offer benefits for young people, particularly by enhancing self-compassion and compassion for others. The observed increase in depressive symptoms in the intervention group, despite improvements in self-compassion, warrants further investigation. Larger studies are needed to confirm these preliminary results and to better understand the underlying mechanisms.

## 1 Introduction

Young people face a variety of daily challenges including physical, cognitive, psychological, and social (Lathren et al., 2019) and have a higher degree of stress, anxiety and depression than at other periods of life (Arnett, 1999; Shorey et al., 2022). These emotional issues among young people have notably increased over the past decades, particularly among females (Shorey et al., 2022; Thapar et al., 2022; Bor et al., 2014), a trend also observed in Sweden (The Swedish National Board of Health and Welfare, 2024; The Public Health Agency of Sweden, 2018) where this study was conducted. Anxiety and depressive disorders are known to be more prevalent in females, with an approximate 2:1 ratio compared with males (Kalin, 2020). Such internalized psychological issues often persist into adulthood if untreated, significantly impacting individuals’ lives (Clayborne et al., 2019; James et al., 2020; Thapar et al., 2012).

Comorbidity among stress, anxiety, and depression is common (Garber and Weersing, 2010; Ehrenreich-May et al., 2017; Kalin, 2020; Vicent et al., 2023), including among young people (Kalin, 2020; Waszczuk et al., 2014). Anxiety and depression are often interwoven rather than distinct constructs across development (McElroy et al., 2018). Evidence-based treatment protocols, particularly those using cognitive behavioral approaches, have been shown to reduce anxiety and depression symptoms in young people (Zemestani et al., 2023; Ehrenreich-May et al., 2017). However, these treatments often focus on symptoms of a single disorder rather than comorbidities or underlying factors (such as self-criticism), leading to suboptimal treatment outcomes (Ehrenreich-May et al., 2017; Wakelin et al., 2022).

Young people’s emotional challenges are frequently linked to concerns about self-evaluation, with negative self-judgment contributing to internal psychological problems (Neff and McGehee, 2010). Shame and self-criticism increase vulnerability and perpetuate issues like stress, anxiety and depression (Gilbert and Procter, 2006). By targeting underlying mechanisms such as emotion dysregulation and negative affect, transdiagnostic treatments can be helpful for those with emotional challenges (Zemestani et al., 2023). Compassion-focused therapy (CFT) is a transdiagnostic approach that targets common psychological processes essential for mental health (Perkins et al., 2023).

The theory of compassion has roots in evolutionary theory, neurobiology, family theory, attachment theory, neuroscience and Buddhist philosophy. CFT contains elements of a variety of therapeutic interventions, including behavior therapy (Gilbert, 2014; Gilbert, 2020). There are several definitions of compassion (Egan et al., 2022; Strauss et al., 2016) and Gilbert’s (2014) definition is “a sensitivity to suffering in self and others, with a commitment to try to alleviate and prevent it” (p. 19). CFT includes psychoeducation on the nature of compassion and Gilbert (2009) suggests that there are three flows of compassion – compassion for self, compassion for others and receiving compassion from others. Low levels of self-compassion and compassion from others have been shown to significantly correlate with both depression and suicidal ideation in adolescents (Jonsson and Dennhag, 2023).

Compassion-focused therapy also involves developing an understanding of the evolved nature of the human mind and body. For example, evolution has left us with “tricky brains” that get caught up in thinking–feeling loops and it has been proposed that human emotions evolved to serve specific functions that are represented by three systems – threat, drive and soothing (Beaumont et al., 2024). The drive system focuses on achieving and activating seeking–engagement strategies; the threat system is threat-focused and its function is to activate strategies that have evolved to keep us safe; and the soothing system is a positive system for well-being that helps to regulate the threat and drive systems (Gilbert, 2014). CFT seeks to increase compassion and activate the soothing system, bringing balance to the threat and drive systems. This approach not only helps young people to cope with current challenges but also prepares them for upcoming stressors and strengthens their willingness to explore (Bluth et al., 2018).

The evidence base for CFT has grown in both clinical and non-clinical populations (Ferrari et al., 2019; Kirby, 2017; Millard et al., 2023; Neff, 2023; Beaumont et al., 2016; Beaumont et al., 2021). Over the past 30 years, studies on CFT have demonstrated benefits for adults, including reductions in depression, self-criticism, distress, and anxiety (Ferrari et al., 2019; Marsh et al., 2018; Williamson, 2020; Craig et al., 2020; Wakelin et al., 2022; Petrocchi et al., 2023).

Despite the importance of intervening during adolescence to prevent the development of psychopathologies (Bluth et al., 2017), relatively few CFT intervention studies have focused on young people (Boggiss et al., 2020; Bluth et al., 2016; Bratt A. et al., 2020; Karr et al., 2020; Seekis et al., 2023). A review by Egan et al. (2022) reported promising outcomes, suggesting that self-compassion interventions can reduce depression and anxiety in young people. However, there remains a need for randomized controlled trials (RCTs) (Bridge et al., 2023), as no RCT has yet evaluated CFT specifically for young people.

Although mental health disorders are highly prevalent in young people (Merikangas et al., 2010; Shorey et al., 2022), most do not receive psychological treatment (Merikangas et al., 2011). In Sweden, access to psychological care varies widely across regions, partly due to availability issues (The Swedish National Board of Health and Welfare, 2019). Internet-based interventions have improved access to effective psychological treatments (Vigerland et al., 2016), with usage growing significantly during the COVID-19 pandemic (Lin et al., 2021). Evidence supports the effectiveness of internet-delivered interventions, including videoconferencing, in improving youth mental health (Porter et al., 2022; von Wirth et al., 2023; Stewart et al., 2017; Karing, 2023; Stewart et al., 2020; Ebert et al., 2015; Välimäki et al., 2017). These interventions may also benefit socioeconomically and digitally marginalized young people (Piers et al., 2023).

Group therapy offers the advantage of reaching multiple young people simultaneously, although some individuals may find it challenging to engage in a group setting (Erekson et al., 2024). Nevertheless, studies suggest that psychotherapy outcomes for young people are comparable in both group and individual formats (Eckshtain et al., 2020). Small group-based CFT with young people has been conducted with positive experiences of the group setting (Bratt A. et al., 2020; Lau-Zhu and Vella, 2023).

This study is part of the project CUST (Compassion-Focused Therapy for Young People with Stress) in northern Sweden, which aims to develop and evaluate internet-based compassion-focused interventions for young people with emotional challenges in rural areas of the country (Dennhag, 2018).

The aim of this study is to determine whether therapist-led internet-based videoconferencing group CFT is feasible and acceptable to use with young people between 15 and 20 years of age in Sweden. Additionally, the study assesses preliminary effects on stress, anxiety and depression symptoms to determine if a larger RCT is justified.

## 2 Methods

**Figure 1:**
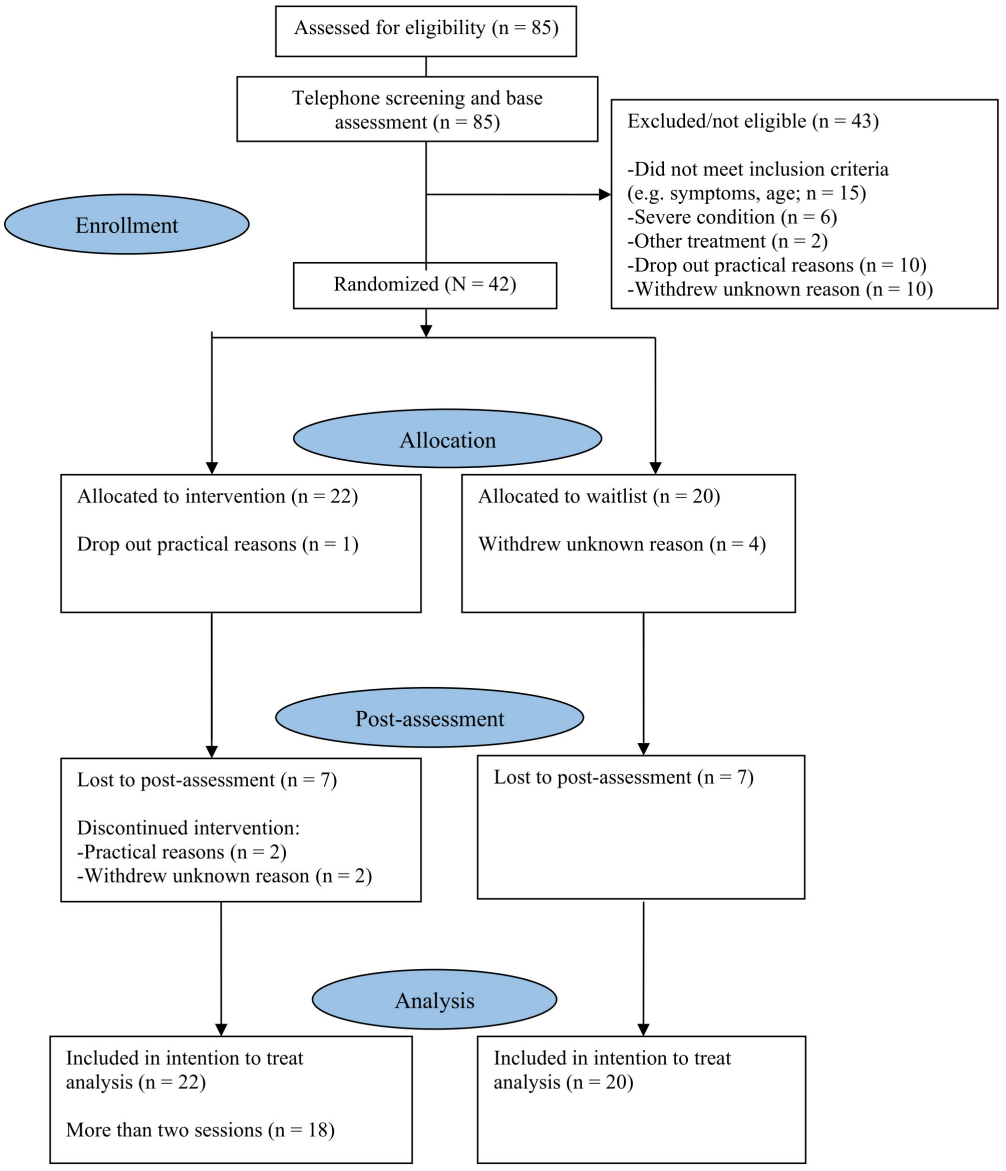
CONSORT flowchart of participants.

This study was a single-blinded, two-arm (CFT intervention group vs. WL group) randomized controlled trial for young people with mild to moderate symptoms of depression, anxiety or stress. The clinical trial protocol was approved by the Swedish Ethical Review Authority (no. 2021–04357; 2022–01318-02, 2022–02931-02) and registered at ClinicalTrials.gov (NCT05448014). Informed consent was obtained from all participants. The study adhered to the CONSORT statement (Eldridge et al., 2016; Butcher et al., 2022) and participant flow is illustrated in Figure 1.

### 2.1 Procedure

A preliminary study was previously conducted with seven participants, six of whom completed the CFT intervention. The results indicated that none of the exercises had a negative impact, and overall, participants reported a positive experience with CFT (Sjölander, 2022).

The current study was promoted to young people via social media, flyers, and direct outreach by project staff or personnel at schools and primary care health centers in northern Sweden. The recruitment efforts were aimed at individuals of all genders. Young people interested in participating contacted a research assistant by text message or email. The research assistants were psychology students with at least 3 years of psychology studies including clinical experience. They administered the assessment procedure, which included web-based questionnaires and a semi-structured phone interview to determine eligibility. The principal investigator (PI; also author Inga Dennhag) regularly supervised the assistants.

Study data were collected and managed using REDCap (Research Electronic Data Capture) hosted at Umeå University (Harris et al., 2009; Harris et al., 2019). REDCap is a secure, web-based platform designed to support data capture for research studies.

A CFT group was initiated once the sufficient number of participants (*n* = 4–8) were available for randomization. During the same period that a CFT group completed the pre- and post-intervention questionnaires, a WL group did the same. To ensure that the intervention could start with enough participants per group within a reasonable time after sign-up, five non-randomized participants who did not meet the inclusion criteria for symptom level were included in the sessions but excluded from the analyses.

In total, seven CFT groups were conducted between September 2022 and January 2024, covering all seasons of the year. The interventions were led by a psychotherapist, supported by an assistant. Across the seven groups, two psychotherapists served as leaders, with the PI being one of them. Both psychotherapists have experience in providing psychotherapy for young people and the PI has additional training in compassion-based approaches for young people. For the first group, the PI led the sessions, with the other psychotherapist assisting. The therapist and assistants received ongoing supervision from the PI.

### 2.2 Participants

Participants invited to this study were between 15 and 20 years old. The inclusion criteria were: (a) symptoms of stress (≥22 on PSS) and/or symptoms of anxiety (≥9 in subscale Anxiety in TSCC) and/or symptoms of depression (≥10 in subscale Depression in TSCC), (b) the ability to speak Swedish, (c) the ability to read and complete forms in Swedish, (d) had at least one close and stable relationship with an adult, and (e) ability to participate in an online group setting. The exclusion criteria included: (a) severe psychological issues that could hinder participation in group treatment, (b) suicidal risk (scoring 4 or higher on item 12 in MADRS-Y self-report with clinical assessment of active suicidal intent during the diagnostic screening interview), (c) bipolar disorder, (d) autism, (e) anorexia nervosa, (f) current substance or alcohol dependence, (g) current psychosis, (h) current active participation in psychotherapy, and (i) recent initiation or withdrawal of antidepressant treatment (participants with prescribed medications for anxiety or depressive disorders were eligible if the dosage had remained constant for at least 1 month).

### 2.3 Randomization

Randomization was performed by a statistician from another department (Registercentrum Norr, Umeå University). Block randomization with a block size of four was used to assign participants to the intervention and WL groups simultaneously. Blinded outcome assessment was applied to minimize bias in estimating treatment effects (Kahan et al., 2014).

### 2.4 Waitlist control group

Participants in the WL group completed self-report questionnaires at pre-intervention and again after 8 weeks (post-intervention). There was no scheduled contact with study staff during the WL period. However, participants had access to contact details to the research assistant and the PI. After completing the WL, participants were offered the CFT intervention. Those who participated in the intervention after the WL period are not included as intervention participants in the analyses.

### 2.5 The CFT intervention

A new Swedish CFT workbook (Dennhag, 2021) was developed specifically for young people in Sweden by the last author, Inga Dennhag, inspired by Professor Paul Gilbert’s CFT model and by the CFT ideas and practices developed for young people in The Kindness Workbook (Beaumont and Welford, 2020). The intervention incorporates key elements of CFT, including the three affect regulation systems—threat, drive, and soothing; the development of a compassionate self; addressing self-criticism, shame, and guilt; practicing mindfulness; and fostering emotional regulation through compassion.

**Figure 2:**
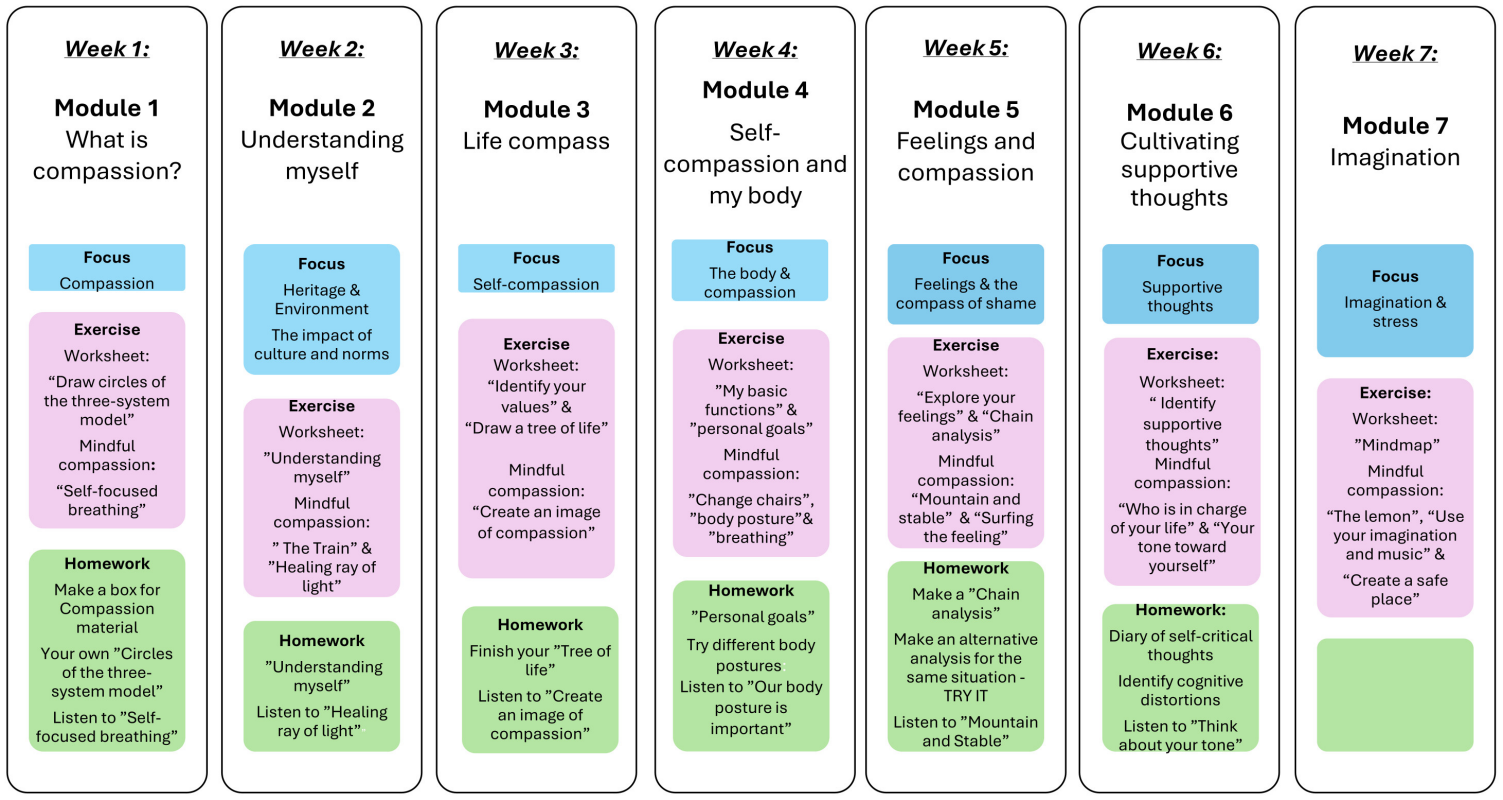
Overview of the CFT intervention structure.

The Swedish workbook (Dennhag, 2021) includes seven modules: (1) What is compassion?, (2) Understanding myself, (3) Life compass, (4) Self-compassion and my body, (5) Feelings and compassion, (6) Creating balanced thoughts, and (7) Imagination. There are a total of 20 exercises. Participants received a digital copy of the workbook (Dennhag, 2021) to practice between sessions, and the leaders had PowerPoint slides and a therapist manual containing the same modules as the workbook. Each session followed a consistent structure, designed to strengthen the participants’ capacity for compassion, alternating between theory and exercises, and concluding with an introduction to the homework assignment. An overview of the intervention content is presented in Figure 2.

### 2.6 Acceptability and feasibility

Total dropout rates, as well as the percentage of completed pre-intervention and post-intervention assessments, were analyzed for both groups. The treatment adherence was assessed by classifying participants who completed fewer than half of the modules (<4) as having discontinued treatment (Grudin et al., 2022). The mean number of completed modules was also calculated.

Participants were asked to complete a written evaluation in which they rated the following: (1) whether the content and objectives were clearly explained, (2) whether the training goals were met, (3) the number of sessions they attended (despite attendance records being maintained), (4) the extent to which they completed the homework assignments, and (5) their overall satisfaction with the training. Additionally, the evaluation invited further feedback on the intervention, including perceived advantages and disadvantages.

Participants were instructed to report any adverse events during the intervention period to the research assistants or group leader. Adverse events were also assessed post-intervention with the question: “Did the CFT training help you to feel better?” with one of the response options being: “No, it seems to have made it worse.”

It was also important to assess whether the CFT leaders found the intervention acceptable and if they were able to deliver it as intended, and to identify any difficulties in implementing program elements or other challenges (Teresi et al., 2022). This was investigated through a feedback survey specifically designed for the psychotherapists and their assistants involved in the CFT treatment, administered after all intervention groups were completed.

### 2.7 Measures

All measures were web-based, self-reported questionnaires administered at pre- and post-intervention.

#### 2.7.1 Primary outcomes

##### 2.7.1.1 TSCC – Trauma Symptom Checklist for Children

The TSCC (Briere, 1996) is a self-report questionnaire for children and adolescents aged 8–17 that measures symptoms related to traumatic experiences. Responses are recorded on a 4-point scale ranging from 0 (“never”) to 3 (“almost all the time”). The internal consistency has shown to be good (Briere, 1996), including for the Swedish translation (Nilsson et al., 2008). TSCC includes six clinical scales with 9–10 items in each. In the current study, the Anxiety and Depression clinical scales were used as primary outcomes, and the Cronbach’s alpha was 0.87.

##### 2.7.1.2 PSS-10 – The 10-item Perceived Stress Scale

The PSS-10 (Cohen S., 1988) is a self-report questionnaire for young people and adults that measures feelings and thoughts related to the stress-associated components of unpredictable, uncontrollable, and overwhelming life events. The self-report is measured on a 5-point scale, ranging from 0 to 4, with higher scores indicating greater levels of stress (Nordin and Nordin, 2013). The PSS-10 has acceptable psychometric properties (Lee, 2012) and its internal consistency has shown to be good, including for the Swedish translation (Nordin and Nordin, 2013). In the current study, the Cronbach’s alpha was 0.85.

#### 2.7.2 Secondary outcomes

##### 2.7.2.1 MADRS-Y – Montgomery–Åsberg Depression Rating Scale – Youth

The MADRS-Y is a self-report questionnaire for young people aged 12 to 20 (Vestin et al., 2022) adapted from the adult version MADRS-S (Svanborg and Åsberg, 1994). This 12-item self-report uses a 7-point scale ranging from 0 (low) to 6 (high), and a higher score indicates more severe depression. MADRS-Y was included in the current study to measure symptoms of depression according to the DSM-5 (American Psychiatric Association, 2013), but as a secondary outcome measure because it has just recently been validated. The internal consistency has been shown to be good in both a Swedish normative sample (Vestin et al., 2022) and a Swedish clinical sample (Vestin et al., 2024). In the current study, the Cronbach’s alpha was 0.82.

##### 2.7.2.2 CEASY-SE – The Compassionate Engagement and Action Scale for Youths – Swedish version

The CEASY-SE is a self-report questionnaire for young people aged 12 to 20, adapted from the adult version CEAS (Gilbert, 2017). It includes the three subscales: compassion for others, compassion from others, and self-compassion (Henje et al., 2020). The 30-item self-report is measured on a 10-point scale ranging from 1 (“never”) to 10 (“always”). The internal consistency has shown to be good in a Swedish sample (Henje et al., 2020). In this study, three control items were removed, resulting in a 27-item version. Each subscale, as well as the total scale, was analyzed separately. In the current study, the Cronbach’s alpha was 0.91 for compassion for others, 0.91 for compassion from others, 0.88 for self-compassion and 0.90 for the total scale.

### 2.8 Statistical analysis

Statistical analyses were conducted using SPSS version 28.0. Data were screened for faulty values and dependent variables were examined for normality. Boxplots were used to check for outliers and the data were examined for normality. Outliers were treated as missing values. We identified only one outlier in the control group, where extreme values (either very low or very high) were recorded at post-intervention. Continuous variables were checked for normality using histograms and scores for skewness and kurtosis. Normal Q–Q plots and scatterplots indicated that the assumptions of linearity and homoscedasticity were satisfied. Independence of observations was assessed with the Durbin-Watson test and all scales were around 2 (ranging from 1.91 to 2.21). There was no multicollinearity in the regression models measured by variance inflation factor (VIF) and tolerance measures.

Descriptives were calculated using standard measures. Sum scores, means, and standard deviations were calculated for each self-report measure.

Missing data were imputed on pre-test using multiple imputation (*n* = 354) missing values of 2,586 (13.69%) on PSS, TSCC, CEASY-SE at pre-test, and *n* = 872 missing values (33.72%) on post-test with 10 iterations and 50 imputations, to enable calculation of sum scores despite the missing items. For the pre-test scores, the imputation model was predicted by the baseline scores of TSCC Anxiety and Depression subscales (complete data), pre-test scores of TSCC, PSS, MADRS-Y, CEASY-SE age, gender, and socioeconomic status, and we used a random seed. For the post-test scores, the imputation model was predicted by the baseline scores of TSCC Anxiety and Depression subscales (complete data) and post-test scores of TSCC, PSS, MADRS-Y, CEASY-SE age, gender, and socioeconomic status were used as predictors, and we used a random seed. WL group and intervention group data were imputed separately. The results from imputed data did not differ from the unimputed data.

We used intention-to-treat analysis (ITT) (Gupta, 2011), meaning that every randomized subject was included in the analyses. We also calculated results for those who participated in more than two intervention sessions. Between-group differences at pre-test between CFT and WL group were calculated with independent *t*-tests.

Between-group differences on primary and secondary outcome measures were calculated with several linear regressions, where post-test values were the dependent variable, and pre-test values and group (intervention or waitlist group) were the independent variables. Within-group differences were calculated with paired sample *t*-tests. Both Cohen’s *d* (using the sample standard deviation of the mean difference) and Hedges’ *g* corrections (using the sample standard deviation of the mean difference plus a correction factor) were used to illustrate effect sizes. We interpreted the within-group differences according to Cohen’s definition of small (*d* ≥ 0.20), medium (*d* ≥ 0.50), and large effects (*d* ≥ 0.80) (Cohen J., 1988). Hedges’ *g* is similar to Cohen’s *d* but includes a correction factor for small samples.

Additionally, within-participant change was calculated using clinically significant change (Jacobson and Truax, 1991; Blampied, 2016). Clinically significant change in stress symptoms (PSS) was computed according to Jacobson and Truax (1991) with two criteria: the change should be reliable and the client should before intervention belong to a dysfunctional domain and after intervention to a functional domain. The cutoff score to separate the dysfunctional domain from the functional domain was computed as c [c = (SD_0_*M_1_ + SD_1_*M_0_)/(SD_0_ + SD_1_) M_0_ = mean of normative sample, SD_0_ = standard deviation of normative sample, M_1_ = mean of patient sample, SD_1_ = standard deviation of patient sample]. The reliable change was calculated with the Reliable Change Index (RCI), as RCI = (X_2_-X_1_)/S_diff_ (S_diff_ = √2(SE)^2^) (SE = SD_1_√1-r, where r is the reliability of PSS; here, *r* = 0.85). The RCI specifies the amount of change a client must show on a specific psychometric instrument between measurement occasions for that change to be reliable. Participants in the intervention group with a clinically significant change, i.e., both a change from a dysfunctional to a functional domain on PSS and a reliable change, were classified as “recovered.” Participants with only a reliable positive change on PSS were classified as “improved.” Participants with no reliable change were classified as “unchanged” and participants with a reliable negative change were classified as “deteriorated.”

## 3 Results

The overarching aim was to determine whether therapist-led internet-based group CFT is feasible and acceptable for treating depression, anxiety and stress in young people aged 15 to 20 in Sweden. An overview of the participant flow is presented in Figure 1. Of the total sample (*N* = 42), 90% of the participants were female and the mean age was 17.2 years (range 15–20 years). For socioeconomic data, the Swedish socioeconomic classification system (SEI) developed by Statistics Sweden (SCB (Statistics Sweden), 1982) was used to estimate a socioeconomic ranking based on six distinct classes. In the current sample, the parents’ socioeconomic status was distributed across only four classes, with no parents classified as unemployed or students. Socio-demographic data did not differ between the intervention and WL groups. The demographic characteristics of the participants are presented in Table 1.

**Table 1:** Baseline demographic and characteristics for each group and total sample.

Baseline characteristic	Intervention group (*n* = 22)		Waitlist group (*n* = 20)		Total sample (*n* = 42)	
	Mean	SD	Mean	SD	Mean	SD
	*n*	%	*n*	%	*n*	%
Age (min-max 15–20)	17.1	1.8	17.4	1.7	17.2	1.7
Gender						
Female	21	95.5	17	85	38	90.5
Male	1	4.5	3	15	4	9.5
Living situation						
With both parents	12	54.5	10	52.6	22	53.7
With one parent	4	18.2	1	5.3	5	12.2
Alternating between parents	3	13.6	1	5.3	4	9.8
Alone	2	9.1	6	31.6	8	19.5
Other	1	4.5	1	5.3	2	4.8
Missing			1			1
Parents socioeconomic status						
Manual workers	3	13.6	4	21.1	7	17.1
Higher civil servants	7	31.8	7	36.8	14	34.1
Self-employed	12	54.5	8	42.1	20	48.8
Missing			1			1
Contact with healthcare in the last year for psychological problems						
Yes	17	77.3	16	80	33	78.6

Valid percent was calculated.

### 3.1 Feasibility and acceptability

Feasibility and acceptability were evaluated based on dropout rates, retention, adherence, participant satisfaction, compliance, and clinician feedback. The dropout rate for the intervention group (*n* = 22) was 23% (*n* = 5), while the dropout rate for the WL group was 20% (*n* = 4). Completion rates for the pre-intervention assessment were 86% overall (*n* = 36), with 91% for the intervention group (*n* = 20) and 80% in the WL group (*n* = 16). Post-intervention assessment rates were 67% overall (*n* = 28), with 68% for the intervention group (*n* = 15) and 65% for the WL group (*n* = 13).

**Figure 3:**
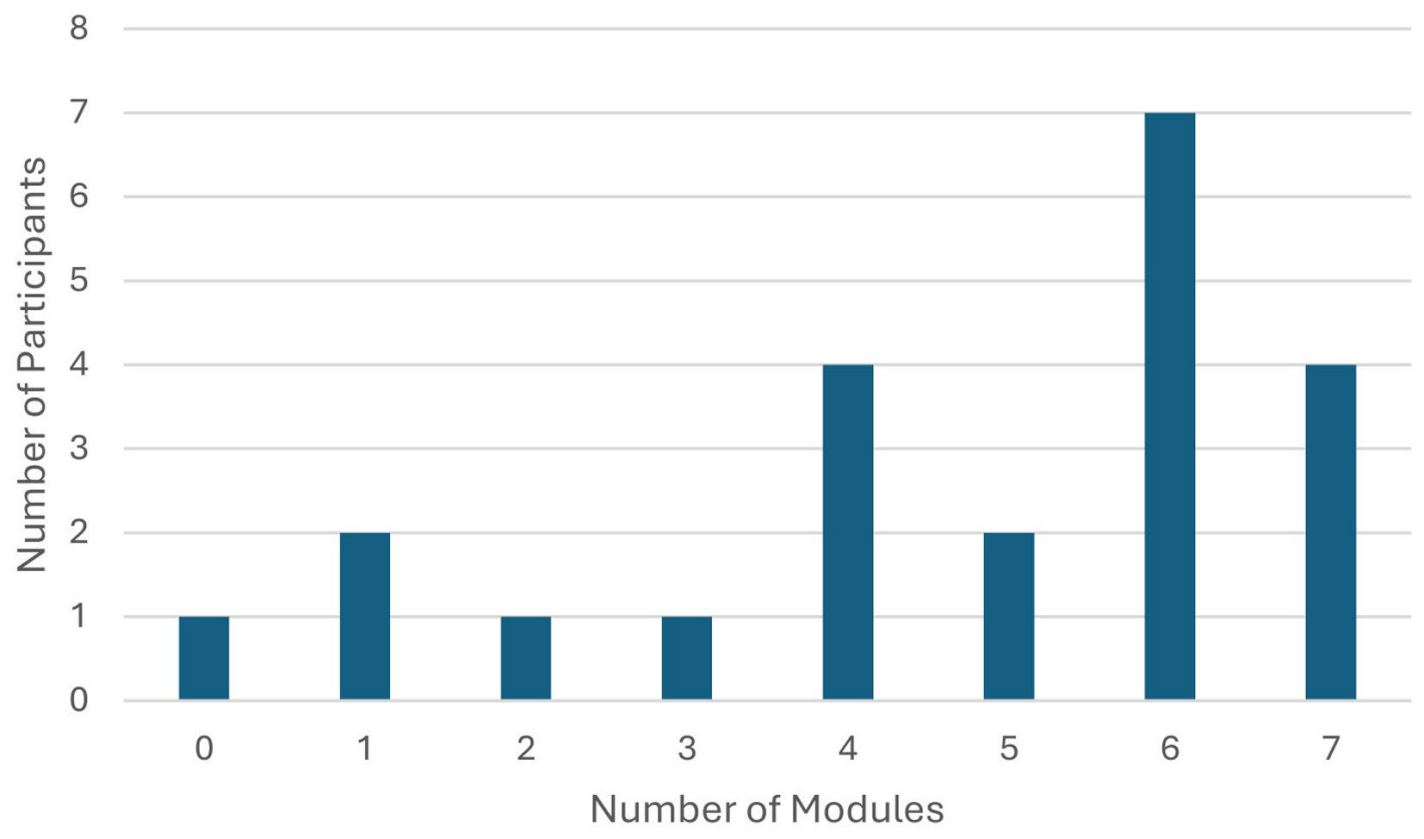
Completed modules during the CFT intervention.

In terms of intervention adherence, participants completed an average of 5.5 modules (*M* = 4.68, *SD* = 2.12) out of seven. A total of 18% completed all the modules, while 82% completed more than two modules. Additionally, 77% completed four or more modules, indicating a moderate level of adherence to the intervention. Dropout tended to occur more commonly early in the intervention process. For a detailed overview (see Figure 3).

After the intervention, participants in the intervention group completed an evaluation form (*n* = 15). Most participants (*n* = 14) felt the intervention’s content and objectives (increasing self-compassion, reducing self-criticism, and improving the regulation of unpleasant emotions) were clearly explained, either completely (*n* = 10) or to some extent (*n* = 4) before the start of the intervention. One participant indicated “did not know.” Most participants (*n* = 13) also felt that the objectives were addressed within the intervention, either completely (*n* = 7) or to some extent (*n* = 6). One participant responded “no,” and one selected “did not know.” Approximately one-quarter of the participants (*n* = 4) reported completing their homework almost always or often, nine completed it sometimes, and two completed it rarely or never. Many participants (*n* = 10) were either very satisfied (*n* = 3) or mostly satisfied (*n* = 7) with the intervention; five participants were neutral, and none were dissatisfied. No adverse events were reported during the intervention, and no participant selected the response “No, it seems to have made it worse” to the question “Did the CFT training help you feel better?”

The psychotherapists and research assistants (hereafter referred to as leaders) who led the groups also completed an evaluation form after the intervention groups were finished (*n* = 8) to provide additional insights on the intervention’s feasibility. Most leaders found it easy (*n* = 5) or very easy (*n* = 2) to implement the CFT intervention according to the protocol, while one leader was neutral. All leaders felt comfortable (*n* = 5) or very comfortable using the protocol and all of them agreed that the intervention was accepted by the young people [well (*n* = 5) and very well (*n* = 3)]. Most leaders found the intervention effective (*n* = 5), two expressed a neutral response, and one answer was missing.

### 3.2 Descriptive statistics

Table 2 provides descriptive statistics showing the observed group means and standard deviations for primary and secondary outcomes at both pre-intervention and post-intervention. There was a difference between the intervention and WL groups in the pre-intervention assessment. The intervention group had higher scores for stress, depression, and anxiety.

**Table 2:** Descriptive statistics for observed group means and standard deviations of primary and secondary outcomes.

Scale	Intervention group (*n* = 22)		Waitlist group (*n* = 20)	
	*n*	*M* (SD)	*n*	*M* (SD)
PSS 10				
Pre	20	25.35 (4.67)	16	21.87 (5.89)
Post	15	22.73 (3.84)	13	20.85 (5.83)
TSCC anxiety				
Pre	19	9.37 (4.98)	16	6.31 (3.00)
Post	15	10.87 (4.96)	13	6.15 (3.93)
TSCC depression				
Pre	19	10.05 (4.26)	16	8.19 (3.10)
Post	15	11.33 (3.64)	13	7.31 (3.43)
MADRS-Y				
Pre	20	28.25 (7.79)	15	21.73 (8.19)
Post	15	26.07 (9.54)	12	16.75 (7.55)
CEASY-SE: compassion for others				
Pre	20	76.15 (9.13)	15	71.47 (13.50)
Post	15	78.20 (6.88)	12	73.42 (16.30)
CEASY-SE: compassion from others				
Pre	20	49.30 (14.77)	15	64.27 (14.58)
Post	15	58.33 (13.71)	12	64.25 (17.66)
CEASY-SE: self-compassion				
Pre	20	43.35 (11.86)	16	46.19 (13.58)
Post	15	52.93 (5.54)	13	48.92 (18.03)

M, mean; SD, standard deviation; Pre- and post-intervention: PSS, Perceived Stress Scale; TSCC, Trauma Symptom Checklist for Children; MADRS-Y, Montgomery–Åsberg Depression Rating Scale – Youth; CEASY-SE, Compassionate Engagement and Action Scales for Youths – Swedish version.

### 3.3 Efficacy of the CTF intervention

The preliminary effects on stress, anxiety, and depression symptoms were calculated and presented as both between-group and within-group differences.

#### 3.3.1 Between-group differences

In the linear regression models for intention-to-treat (ITT) analyses, two variables showed significant differences between the intervention and WL groups at the *p* < 0.05 level. An unexpected finding in the primary outcome was that depression (TSCC Depression subscale) had significantly increased in the intervention group compared to the WL group. This result indicates that depression symptoms deteriorated more in the intervention group than in the WL group. For the secondary outcomes, self-compassion (CEASY-SE) increased significantly in the intervention group and differed significantly from the WL group. Table 3 presents the results of the regression analysis of associations between groups for the ITT.

**Table 3:** Regressions of associations between intervention and waitlist groups.

Variable	*B*	*SE*	*t*	*p*	95% CI
Primary outcome					
PSS					
Groups (intervention vs. waitlist)	0.06^a^	1.11	0.46	0.652	−1.74 – 2.75
TSCC anxiety					
Groups (intervention vs. waitlist)	−0.25^a^	1.14	−1.77	0.080	−4.34 – 0.26
TSCC depression					
Groups (intervention vs. waitlist)	−0.44^a^	0.88	−3.32	0.002**	−4.69 – −1.14
Secondary outcome					
MADRS-Y					
Groups (intervention vs. waitlist)	−0.21^a^	2.16	−1.55	0.130	−7.69 – 1.02
CEAS-Y-SE compassion for others					
Groups (intervention vs. waitlist)	−0.14^b^	2.75	−1.02	0.315	−8.35 – 2.76
CEAS-Y-SE compassion from others					
Groups (intervention vs. waitlist)	−0.30^b^	3.91	−1.91	0.064	−15.37 – 0.45
CEAS-Y-SE self-compassion					
Groups (intervention vs. waitlist)	−34^b^	2.98	−2.36	0.023*	−13.05 – −1.00

Intention to treat group (*n* = 22) compared to waitlist group (*n* = 20).

CI, confidence interval; B, standardized beta values.

^aA positive value of standardized beta values for groups indicates a greater reduction of symptoms in the intervention group.^

^bA negative value of standardized beta values for groups indicates a greater increase of compassion in the intervention group.^

PSS, Perceived Stress Scale; TSCC, Trauma Symptom Checklist for Children; MADRS-Y, Montgomery–Åsberg Depression Rating Scale – Youth; CEAS-Y-SE, Compassionate Engagement and Action Scales for Youth – Swedish version.

*p < 0.05, **p < 0.01, ***p < 0.001.

A pattern similar to the ITT analyses was observed in the analyses based on participation in more than two sessions. Depression (TSCC Depression subscale) significantly increased in the intervention group compared to the WL group. Self-compassion also significantly increased in the intervention group compared to the WL group. See Table 4 for the results.

**Table 4:** Regressions of associations between intervention and waitlist groups values for participants completed more than two sessions.

Variable	*B*	*SE*	*t*	*p*	95% CI
Primary outcome					
PSS					
Groups (intervention vs. waitlist)	0.05^a^	1.18	0.36	0.725	−1.98 – 2.82
TSCC anxiety					
Groups (intervention vs. waitlist)	−0.19^a^	1.24	−1.34	0.190	−4.17 – 0.86
TSCC depression					
Groups (intervention vs. waitlist)	−0.42^a^	0.97	−2.96	0.005**	−4.83 – −0.90
Secondary outcome					
MADRS-Y					
Groups (intervention vs. waitlist)	−0.19^a^	2.31	−1.34	0.190	−7.78 – 1.60
CEAS-Y-SE compassion for others					
Groups (intervention vs. waitlist)	−0.15^b^	3.04	−1.05	0.302	−9.37 – 2.99
CEAS-Y-SE compassion from others					
Groups (intervention vs. waitlist)	−0.27^b^	4.14	−1.71	0.097	−15.46 – 1.34
CEAS-Y-SE self-compassion					
Groups (intervention vs. waitlist)	−0.31^b^	3.26	−2.08	0.045*	−13.40 – −0.16

For those who participated in more than two sessions (*n* = 18) compared to waitlist (*n* = 20).

CI, confidence interval; B, standardized beta values.

^aA positive value of standardized beta values for groups indicates a greater reduction of symptoms in the intervention group.^

^bA negative value of standardized beta values for groups indicates a greater increase of compassion in the intervention group.^

PSS, Perceived Stress Scale; TSCC, Trauma Symptom Checklist for Children; MADRS-Y, Montgomery–Åsberg Depression Rating Scale – Youth; CEAS-Y-SE, Compassionate Engagement and Action Scales for Youth – Swedish version.

*p < 0.05, **p < 0.01, ***p < 0.001.

#### 3.3.2 Within-group differences

Paired sample *t*-tests were conducted to evaluate within-group differences among the intervention participants who attended more than two sessions. Significant moderate within-group differences were found for perceived stress (*p* = 0.012), compassion from others (*p* = 0.013), and self-compassion (*p* = 0.006). No statistically significant differences were observed for anxiety or depression symptoms or compassion for others. Table 5 presents an overview of the results.

**Table 5:** Within-group differences between participants in the intervention group who completed more than two sessions (*n* = 18).

Scale	Change mean (95% CI)^a^	*t*(17)	*p*-value (two-sided)	Cohen’s *d*	Hedges’ correction *g*
PSS	2.01 (0.51–3.51)	2.83	0.012*	0.67	0.64
TSCC anxiety	−0.75 (−3.23–1.72)	−0.64	0.530	−0.15	−0.14
TSCC depression	−1.02 (−3.43–1.38)	−0.90	0.381	−0.21	−0.20
MADRS-Y	1.78 (−2.21–5.77)	0.94	0.359	0.22	0.21
CEAS-Y-SE compassion for others	−1.56 (−6.15–3.03)	−0.72	0.483	−0.17	−0.16
CEAS-Y-SE compassion from others	−8.01 (−14.10 – −1.91)	−2.77	0.013*	−0.65	−0.62
CEAS-Y-SE self-compassion	−8.35 (−13.97 – −2.73)	−3.13	0.006**	−0.74	−0.71

^a^Difference of rate of change (change mean): a positive value indicates a greater reduction of symptoms in the intervention group. A negative change in the mean value of compassion indicates a greater increase of compassion.

PSS, Perceived Stress Scale; TSCC, Trauma Symptom Checklist for Children; MADRS-Y, Montgomery–Åsberg Depression Rating Scale – Youth; CEAS-Y-SE, Compassionate Engagement and Action Scales for Youth – Swedish version.

*p < 0.05, **p < 0.01, ***p < 0.001.

#### 3.3.3 Response and deterioration rates

Clinically significant change analyses were conducted for participants (*n* = 18) in the intervention group who completed more than two sessions to assess whether they had recovered, improved, deteriorated, or remained unchanged regarding stress symptoms (PSS). The PSS was chosen because most participants identified themselves as stressed, regardless of depression or anxiety symptoms. The comparative normative Swedish sample for the PSS was aged 18–34 [*N* = 706, 441 women; (Nordin and Nordin, 2013)]. The mean PSS score in the normative sample (M_0_) was 15.6 (SD_0_ = 6.67), whereas the mean in the intervention group (M_1_) was 25.35 (SD_1_ = 4.67). The cutoff score between a functional and dysfunctional domain was estimated at 21.62 on the PSS, with scores equal to or above the cutoff indicating the dysfunctional domain.

Reliable Change Index (RCI) was calculated to determine whether participants in the intervention group demonstrated a statistically reliable change. An RCI greater than 1.96 (*p* < 0.05) indicated a real change, and in our sample, participants needed to decrease their PSS scores by at least ≥1.02 (z) scores to achieve a reliable change. On the PSS scale, a score of 21.62 serves as the cutoff between the functional and dysfunctional domains, with the dysfunctional domain encompassing scores above this threshold.

**Figure 4:**
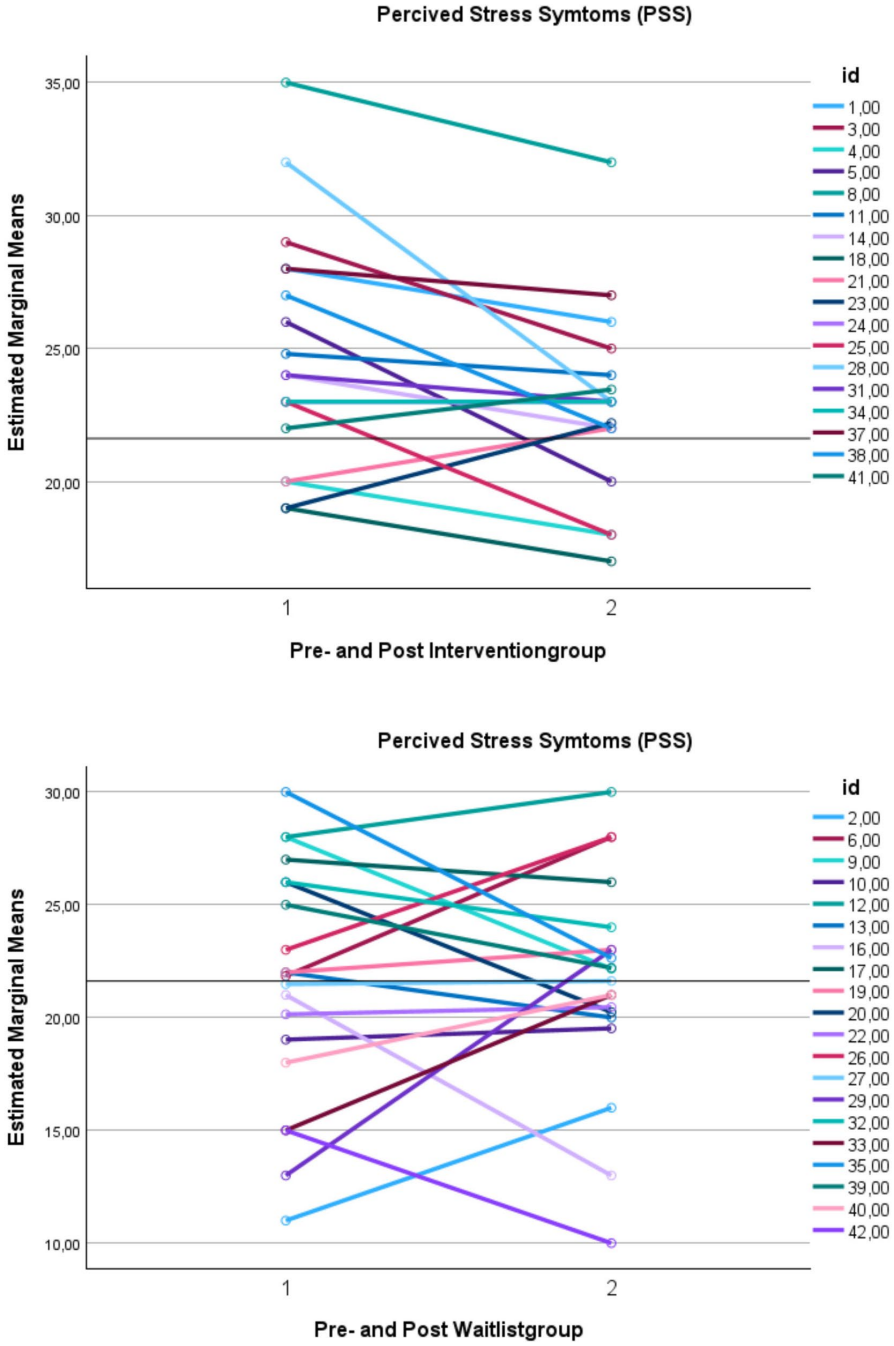
Changes in perceived stress symptoms for participants who attended more than two sessions and waitlist group. This figure demonstrates the means of perceived stress (PSS) over time. PSS, Perceived Stress Scale. *Y*-axis: Means. *X*-axis: Time 1 = scores before intervention, and Time 2 = scores after treatment. A cutoff of 21.62 separates functional and dysfunctional domains; scores above represent the dysfunctional domain, while scores below represent the functional domain.

**Figure 5:**
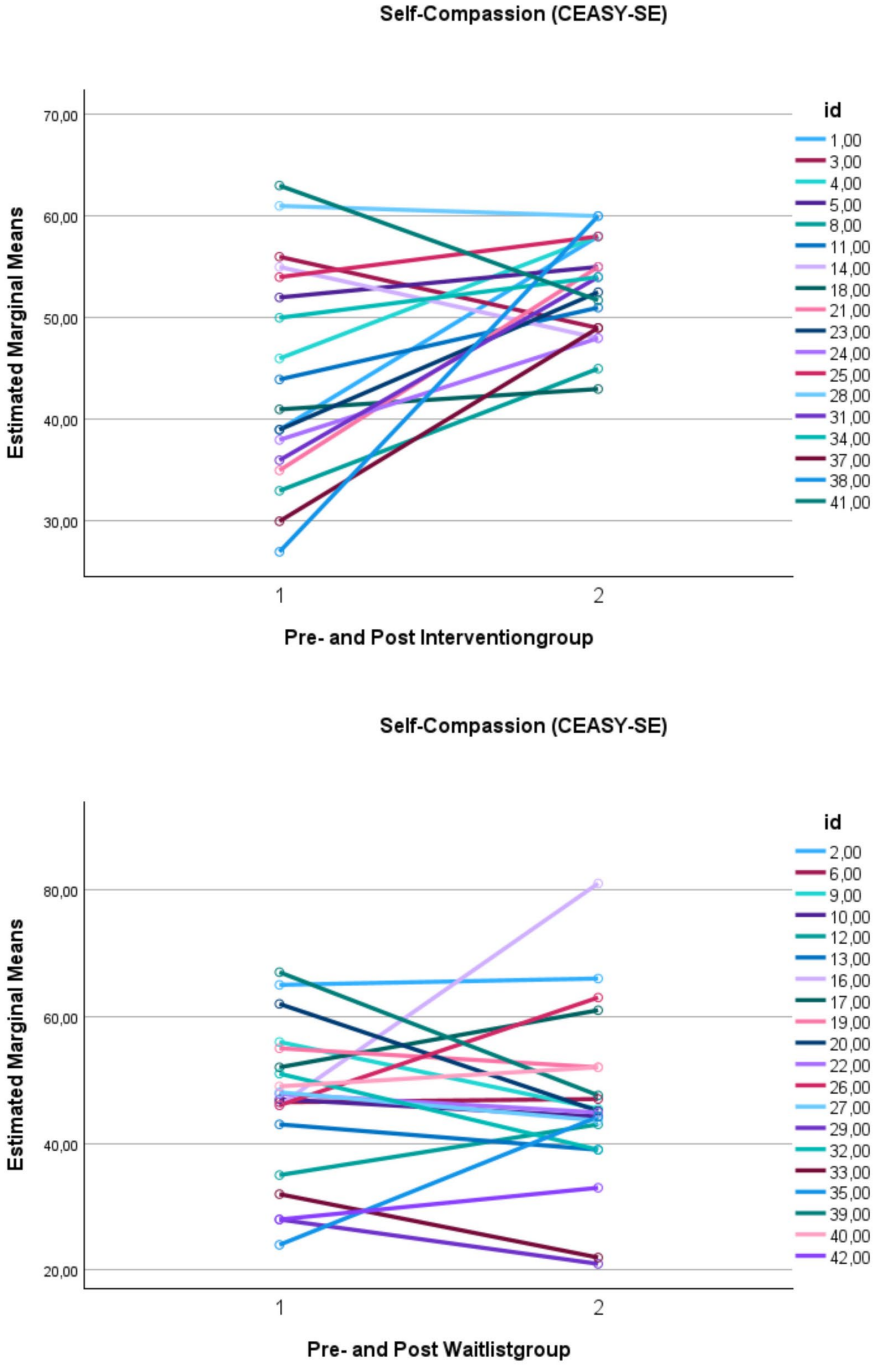
Changes in self-compassion for participants who attended more than two sessions and waitlist group. This figure demonstrates the means of self-compassion (CEAS-Y-SE Self-compassion) over time. CEAS-Y-SE, Compassionate Engagement and Action Scales for Youth – Swedish version. *Y*-axis: means. *X*-axis: Time 1 = scores before intervention, and Time 2 = scores after intervention.

The classification of clinical change indicated that just over three-quarters (78%; n = 14) of all participants were in the dysfunctional domain before therapy. The total outcome, considering a clinical change and a reliable change for PSS, showed that 56% (*n* = 10) responded positively (either recovered 11%; *n* = 2 or improved 44%; *n* = 8), 28% (*n* = 5) remained unchanged, and 17% (*n* = 3) in the intervention group deteriorated. See Figure 4 for the individual means of PSS score before and after group intervention and WL. Figure 5 illustrates individual self-compassion scores before and after the intervention for participants who completed more than two sessions and WL group. As seen in the figure, self-compassion displayed a positive trend in the intervention group.

## 4 Discussion

This study aimed to evaluate the feasibility, acceptability, and preliminary efficacy of a new internet-based, videoconferencing group CFT intervention for young people reporting symptoms of stress, depression, and anxiety. The literature in this area remains limited, and to our knowledge, this is the first RCT conducted with a young population. Therefore, this study is an important contribution to research.

### 4.1 Feasibility and acceptability

Feasibility and acceptability were assessed using a pilot RCT design. The study indicated feasibility and acceptability through low dropout rates, moderate retention and adherence, participant satisfaction, compliance, and positive clinician feedback. Regarding attrition and attendance in the intervention group, 77 % (*n* = 17) completed four or more modules, and 18 % (*n* = 4) completed all seven modules. This indicates moderate participant retention and adherence, which is consistent with findings from other studies (Berg et al., 2022; Grudin et al., 2022). In psychological interventions, dropout rates are often high (Biswal et al., 2024), although it is difficult to estimate the dropout rate specifically in young people receiving therapy for depression due to the inconsistency in how dropout has been reported (O'Keeffe et al., 2019). Dropout rates are suggested to be particularly high when defined by the number of attended sessions, as in the current study, compared to definitions based on the therapist’s assessment (de Haan et al., 2013). We believe that attendance might have been higher if some groups had not been scheduled close to vacations and important school-related events (e.g., student parties). Dropout in psychotherapy is generally challenging among young people due to practical barriers, dissatisfaction, and perceived lack of benefits (O'Keeffe et al., 2019; Ogrodniczuk et al., 2005). This study’s relatively low dropout rate (22%) in the intervention group could be attributed to several factors. The therapist-led and internet-based design minimizes accessibility barriers to evidence-based treatment, such as distance and time conflicts with school and work. The young people in the study appeared to find it easy to attend online using only their smartphones or computers. This finding suggests that the internet-based videoconferencing design may help reduce dropout rates, aligning with other studies (Sibinga et al., 2008; Ahola Kohut et al., 2020).

Most participants felt that the intervention’s content and objectives were well explained, a factor known to reduce dropout (Ormhaug and Jensen, 2018; Timulak and Keogh, 2017; Persson et al., 2017; Watsford and Rickwood, 2014). No harm to participants was reported.

In a previous study (Bratt A. et al., 2020), the essential meaning of group-based CFT was described as *“gaining the courage to see and accept oneself through meeting with peers who are experiencing similar difficulties*” (p. 914). Feeling connected and not being alone is an important part of the journey to approach self-compassion, and a group format might be a helpful contribution. However, the group format for CFT is relatively new and unexplored (Erekson et al., 2024), and more research is needed. Successful therapeutic group treatment requires not only knowledge of CFT but also knowledge of group theory (Bratt A. et al., 2020; Erekson et al., 2024), which was evident in this study.

### 4.2 Preliminary efficacy

Another aim of this study was to assess the intervention’s preliminary effects on stress, anxiety, and depression both between the intervention and the WL groups, as well as within the intervention group. The goal of our intervention was to increase self-compassion, reduce self-criticism, and improve the regulation of unpleasant emotions to affect symptoms of stress, anxiety, and depression. In previous studies, correlations between self-compassion, self-criticism, stress, anxiety and depression are well-established (Vidal and Soldevilla, 2023; Millard et al., 2023). The primary outcomes showed no significant differences between intervention and WL groups for stress and anxiety, a finding identified in previous research (Bratt A. S. et al., 2020).

Surprisingly, depression significantly increased for the intervention group (*p* = 0.05) compared to earlier findings in other studies (Han and Kim, 2023; Egan et al., 2022). However, the findings must be interpreted with caution due to the small sample size. A possible explanation might be that in CFT and our intervention, we practice identifying one’s suffering and daring to be touched by it. The transdiagnostic approach and the intervention’s focus on cultivating a compassionate mind reduce shame and self-criticism (Erekson et al., 2024; Gilbert, 2014), which is crucial for well-being (Marsh et al., 2018; Han and Kim, 2023). The young participants might lack awareness of their degree of self-criticism. This process can be painful and increase grief as participants become more able to see, recognize, and admit suffering (Gilbert, 2010, 2017). The participants with increased symptoms of depression might have identified and experienced emotional pain and would potentially have benefited from additional CFT sessions (Asano et al., 2022). Other CFT group treatments for depression have typically included more sessions (usually around 12) (Asano et al., 2022), with a focus on symptoms of shame and self-critical judgment, in addition to cultivating the flow of compassion.

The secondary between-group outcome revealed increased levels of self-compassion, and the study provides preliminary support for significant positive differences between the intervention group and WL group in self-compassion. This result aligns with similar findings for adults (Asano et al., 2022) that self-compassion increased after CFT group treatment compared to WL.

The within-group results showed a moderately difference in stress, compassion from others, and self-compassion, but not for compassion for others, anxiety and depression. Increased self-compassion aligns with previous research in CFT, both individually (Egan et al., 2022; Marsh et al., 2018) and in groups (Erekson et al., 2024; Lucre and Corten, 2013; Millard et al., 2023; Asano et al., 2022).

Classifying each participant’s change as reliable may complement the preliminary effect sizes for researchers and practitioners by providing a more nuanced understanding of the meaningful change at an individual level (Blampied, 2022). The findings from the study suggest a trend in which participants’ stress levels were reduced, and self-compassion increased after completing the intervention, suggesting potential beneficial effects. These are important findings, particularly since this is a group therapy setting. Previous studies with young people show clear links between higher self-compassion and well-being (Bluth and Blanton, 2015; Bluth et al., 2018; Marshall et al., 2015).

Another noteworthy observation is the gender gap among the participants. The study included around 90 % female participants (95.5% in the intervention group and 85% in the WL group), which is higher than in other studies with predominantly female participants (Millard et al., 2023; Marquez, 2024). However, research suggests that boys of all ages are less likely to seek help for mental health issues than girls of the same age. In a recently published review, self-compassion was found to reduce stigma about mental health, which was found to be a barrier for boys (Sheikh et al., 2024). This indicates that CFT group interventions could be considered in the development of future strategies to reduce barriers to help-seeking for boys.

### 4.3 Limitations

This study has several limitations. It was a pilot RCT feasibility study with a small sample size and preliminary efficacy results. Most participants were female, which reflects the clinical reality but may introduce a gender-based bias. A larger sample size in a longitudinal study with greater statistical power is needed to estimate the intervention’s effects on pre-, post, and follow-up group therapy outcomes.

Feasibility and acceptability were based on self-reporting and retention rates. Acceptability is a multifaceted construct, and a limitation may arise from the operational definitions used in this study. In-depth interviews with the participants could have enhanced the results, providing a stronger basis for understanding prospective and retrospective acceptability.

A limitation of this study is that homework completion was not systematically recorded after each session, even though participants rated the extent to which they completed the homework assignments after the entire treatment. If actual completion had been systematically recorded, it might have provided additional insights into intervention adherence and engagement.

A further limitation was the study design. The design included the primary outcomes of stress, depression, and anxiety symptoms, which was a broad approach with challenges. The study would possibly have benefitted from having self-compassion as the primary outcome, along with stress. Nonetheless, earlier research suggests that CFT improves various clinical symptoms (Millard et al., 2023).

Finally, while participants were randomly allocated to each group, the intervention group presented more severe symptoms of stress, anxiety, and depression and lower levels of self-compassion, which could have created an imbalance. Additionally, fewer participants in the WL group completed post-intervention measures than in the intervention group. However, recommended randomization techniques for small samples (e.g., block randomization, blinded outcome assessment) were used to ensure balance between groups, suggesting that bias should be minimal (Kahan et al., 2014; Netz et al., 2019). Randomization provides each participant an equal chance of assignment, thus helping to make the groups comparable.

### 4.4 Future research

The research team plans to publish a qualitative study exploring focus group findings, which will examine participants’ feedback about the acceptability and effects of the CFT group intervention. Following necessary adaptions, a larger full-scale, mixed-method, longitudinal RCT study could be explored using a comprehensive CFT program.

### 4.5 Conclusion

The findings from this study indicate that the internet-based videoconferencing group CFT intervention is feasible and acceptable for reducing stress and increasing levels of compassion in young people aged 15–20 in Sweden. The preliminary between-group results show that the intervention increased self-compassion and depression. Further research using the CFT intervention over a longer period would help us explore if this would reduce symptoms of depression. The current study suggests that a 7-week module was insufficient time to instigate change. The preliminary within-group results indicate a significant moderate decrease in stress, along with a moderate increase in compassion from others and self-compassion. However, the CFT intervention is promising for preventing stress and increasing self-compassion for young people.

## Data Availability

The raw data supporting the conclusions of this article will be made available by the authors, without undue reservation.
